# Continuous and High-Intensity Interval Training: Which Promotes Higher Pleasure?

**DOI:** 10.1371/journal.pone.0079965

**Published:** 2013-11-26

**Authors:** Bruno R. R. Oliveira, Fabian A. Slama, Andréa C. Deslandes, Elen S. Furtado, Tony M. Santos

**Affiliations:** 1 Exercise and Sports Sciences Graduate Program of Gama Filho University, Rio de Janeiro, Brazil; 2 Physical Education Department of President Antônio Carlos University, Minas Gerais, Brazil; 3 Physical Education Department of Gama Filho University, Rio de Janeiro, Brazil; 4 Physical Education Department of Pernambuco Federal University (Universidade Federal de Pernambuco), Pernambuco, Brazil; Universidad de Granada, Spain

## Abstract

**Objectives:**

To compare the psychological responses to continuous (CT) and high-intensity interval training (HIT) sessions.

**Methods:**

Fifteen men attended one CT session and one HIT session. During the first visit, the maximum heart rate, VO_2Peak_ and respiratory compensation point (RCP) were determined through a maximal cardiopulmonary exercise test. The HIT stimulus intensity corresponded to 100% of VO_2Peak_, and the average intensity of both sessions was maintained at 15% below the RCP. The order of the sessions was randomized. Psychological and physiological variables were recorded before, during and after each session.

**Results:**

There were no significant differences between the average percentages of VO_2_ during the two exercise sessions (HIT: 73.3% vs. CT: 71.8%; p = 0.779). Lower responses on the feeling scale (p≤0.01) and higher responses on the felt arousal scale (p≤0.001) and the rating of perceived exertion were obtained during the HIT session. Despite the more negative feeling scale responses observed during HIT and a greater feeling of fatigue (measured by Profile of Mood States) afterwards (p<0.01), the physical activity enjoyment scale was not significantly different between the two conditions (p = 0.779).

**Conclusion:**

Despite the same average intensity for both conditions, similar psychological responses under HIT and CT conditions were not observed, suggesting that the higher dependence on anaerobic metabolism during HIT negatively influenced the feeling scale responses.

## Introduction

A well-established inverse relationship exists between physical exercise and chronic disease [1], and it has been shown that continuous training (CT) has important health benefits [2]. On the other hand, high-intensity interval training (HIT) has been shown to be ideal for achieving greater improvements in physiological variables because it allows individuals to perform activities at high intensities for longer periods of time [3]. The main difference between these training methods is that CT is characterized by submaximal intensities for prolonged durations and is performed continuously, whereas HIT is characterized by repeated bouts of short-to-moderate duration (i.e., 10 seconds to 5 minutes) at intensities above the lactate or ventilatory threshold [4]. Despite wealth of available knowledge regarding the physiological responses and training effects of each method, little is known about the acute psychological responses of individuals during HIT, which is an important factor for decision-making in exercise prescription. For example, perceived pleasure has been reported to be an important contributor to exercise adherence [5]. It has previously been demonstrated that an increase in perceived pleasure (reported as a shift of one unit on the Feeling Scale) is associated with an additional 38 minutes of physical activity per week [6].

Previous studies have reported an inverse relationship between exercise intensity and psychological responses in CT [5]. However, the effects of HIT sessions on psychological responses are still not known. To the best of our knowledge, only two studies have investigated the effects of CT and HIT on psychological variables [7], [8]. Muller et al. [8] found positive responses in mood after a CT session, whereas no changes were found in mood responses after a HIT session. However, that study was conducted under cold environmental conditions. Bartlett et al. [7] reported higher enjoyment after HIT. It is important to consider that this study did not use a stimulus intensity of 100% of VO_2Peak_, which seems to be the optimal intensity for the acquisition of physiological benefits [3]. In both studies [7], [8], the psychological responses were elicited before and after exercise but not during, as has been previously recommended [9]. No study to date has performed a psychological comparison between HIT and CT during exercise sessions.

According to the Dual-Mode Theory, an exercise session can have effects on cognitive factors (e.g., psychological factors such as physical self-efficacy) and interoceptive factors (e.g., physiological factors such as ventilation), and the interaction of these factors is influenced by the exercise intensity [10]. This theory postulates that the exercise intensity (especially the lactate and ventilatory threshold) is the mediator of affective responses. It has been hypothesized that cognitive factors predominate at intensities below the metabolic threshold and that interoceptive cues predominate at intensities above the metabolic threshold [11]. The predominance of cognitive factors is related to pleasurable sensations, whereas the predominance of interoceptive cues is related to unpleasant sensations [11]. Therefore, stimulation intensities at 100% of VO_2Peak_ would most likely result in negative pleasure; however, it is possible that recovery periods can reduce the negative effects of these stimulation periods.

The studies of Muller et al. [8] and Bartlett et al. [7] investigated specific emotions (mood and enjoyment). However, the affective valence (pleasure and displeasure) and activation (high arousal and low arousal) that comprise basic affect were not included in their investigations. In a conceptual paper, Ekkekakis and Petruzzello [9] recommended that descriptive studies should focus on basic affect and that both categorical (quantitative differences) and dimensional (map of the entire affective space) analyses are compatible and not mutually exclusive. In this context, a dimensional analysis of basic affect using the circumplex model may indicate more completely the effects of each type of training (HIT and CT) on affective responses (valence and activation). Based on these recommendations, we decided that it would be more suitable to analyze basic affect in conjunction with a specific emotion.

Given its superior physiological benefits [12], [13], HIT should be recommended for the improvement of physical fitness. However, the effects of HIT on psychological variables are not well understood, especially with respect to basic affect. Considering that positive psychological responses may improve the likelihood of adherence, it is necessary to investigate which training method (CT or HIT) might result in better psychological responses. Therefore, the aim of this study was to compare the psychological responses to CT and HIT sessions. Considering the recovery period between each HIT stimulus, and the equivalent average intensity between HIT and CT we hypothesized that HIT and CT would result in similar responses.

## Materials and Methods

### Ethics statement

All of the participants signed a written consent form, and the research was approved by the institutional ethics committee of Gama Filho University (#101.2011).

### Participants

Fifteen men from a university community in Rio de Janeiro were personally invited to participate in this study. Their characteristics are reported in Table 1. To determine the sample size [14], we assumed a mean difference of 1.74±1.18 on the Feeling Scale (FS) with an α = .05 and a β = .20 because similar results had been previously observed and were associated with significant differences [15]. The estimated sample size was nine participants; however, we included six additional participants due to the possibility of the population being misrepresented [14]. We included participants aged 18 to 45 years old who were classified as being at low risk for cardiovascular disease [1], and none of the participants had a diagnosis of any mental disorder. Participants who were injured, had a resting blood pressure above 139/89 mmHg, or were not able to perform the time limit test at 100% of VO_2Peak_ for at least four minutes were excluded.

**Table 1:** Participant characteristics.

Variables	M	SD	Min	Max
Age (years)	24	4	18	33
Height (cm)	178.2	7.6	167.5	196.5
Body mass (kg)	76.7	9.4	65.0	101.0
Body mass index (kg.m^−2^)	24.2	2.5	19.8	28.1
% body fat	10.8	4.5	4.7	19.4
RCP* (% VO_2Peak_)	80.3	4.5	72.0	85.0
T_Lim_ (min)	5.12	.86	4.0	6.83
VO_2Peak_ (mL.kg^−1^.min^−1^)	47.9	7.4	35.6	58.7

Note - *respiratory compensation point.

### Experimental Design

On the first visit, the participants signed a consent form and completed a risk stratification questionnaire. We recorded blood pressure and resting heart rate (HR) measurements. Anthropometric measurements were taken, and the participants were instructed regarding the questionnaires and scales to be applied in the study. A maximal treadmill test was performed to determine the peak oxygen consumption (VO_2Peak_), maximum HR and respiratory compensation point (RCP). After a 15-minute recovery period, the participants underwent a time limit test at 100% of VO_2Peak_. During two subsequent visits, HIT and CT sessions on a treadmill were administered in a cross over design with a counterbalanced order. For both conditions (HIT and CT), physiological and psychological measurements were recorded before, during, and after the exercise sessions. An interval of two to seven days between visits was adopted. The participants were instructed not to consume drugs or perform any exercise within 24 hours of the laboratory testing.

### Procedures

#### Anthropometry

Measurements of mass and height were performed (Filizola 31, Filizola S.A., São Paulo, Brazil) to determine the body mass index. The skinfold thickness was measured (Slim Guide, Rosscraft Innovations Inc., Vancouver, Canada) according to the protocol proposed by Jackson and Pollock [16] to determine the body fat percentage [17]. All procedures were supervised by an ISAK level 3 anthropometrist.

#### Psychological measures

To determine perceived exertion, the CR10 scale (RPE) [18] was used. The CR scale was adopted because it has been previously demonstrated that psychophysical ratio scales provide more accurate growth functions (positively accelerating with power output), which allows a better understanding of physiological and perceptual processes, when compared to the 6–20 RPE scales [19]. The FS was used to measure the affective valence (pleasure and displeasure), ranging from −5 (*Very bad*) to +5 (*Very good*) [20]. The perceived activation was measured using the Felt Arousal Scale (FAS), ranging from 1 (*Low arousal*) to 6 (*High arousal*), that was proposed by Svebak and Murgatroyd [21]. The participants were asked to respond to all of the scales according to their feelings at the current moment. To measure specific emotions, we used the profile of mood states (POMS) questionnaire to quantify total mood disturbance (TMD) based on five negative factors, tension, hostility, fatigue, confusion and depression, and one positive factor, vigor [22]. We also used the physical activity enjoyment scale (PAES) to assess the enjoyment level for each exercise condition [23].

#### Maximal exercise testing

The participants performed a maximal exercise test on a treadmill to determine the maximum HR, respiratory compensation point (RCP; as proposed by Beaver et al. [24]), and VO_2Peak_ (determined as the highest value observed during the test). After a 5-minute warm-up at 5 km·h^−1^, the speed was adjusted to 8.5 km·h^−1^ and maintained for three minutes to stabilize the metabolic demand for the running motor pattern. We increased the speed by 1.5 km·h^−1^ every two minutes. Upon reaching 16 km·h^−1^, the speed was stabilized, and the slope was increased by 2% every two minutes until the participants experienced volitional exhaustion. The increase in the slope was used because 16 km·h^−1^ was the maximum speed of the treadmill used in the present study. We used a gas analyzer (Cortex Metalizer II, Cortex Biophysik GmbH. Leipzig, Germany) during the test to record gas exchange variables. The equipment was calibrated before each test based on the manufacturer's instructions. Each participant's heart rate was continuously recorded using a heart rate monitor (RS800CX, Polar Electro OY, Kempele, Finland) during the testing. After a 15-minute recovery period in a sitting position, the participants underwent a time limit test at 100% of VO_2Peak_ to determine their individual capacities to sustain this intensity.

#### Training sessions

The training sessions were applied in a random order during the second and third visits. We used an average intensity of 85% of the RCP for both conditions, and the session duration was determined based on the study by Santos et al. [25] and was set at 50% of the recommended duration. In the HIT sessions, the individuals maintained an intensity of 100% of VO_2Peak_ for two minutes, and recovery was maintained at an intensity of 0%; the recovery duration was adjusted to maintain the same average intensity as the average intensity that was applied in the CT condition. The intensity was determined based on an equation adapted from the proposed method of Saltin et al. [26], described here as Equation 1. Although previous studies have already demonstrated that there is no effect of duration on affective responses [27], the number of HIT stimuli was adjusted to allow the same total duration as the CT sessions. The ambient temperature was set at approximately 20°C. On both visits, the HR and gas exchange variables were recorded continuously. The RPE, FS and FAS scores were elicited after each HIT stimulus, whereas during the CT session, measurements were made at the same time intervals as the HIT session. Following a similar methodological approach used in previous studies [7], [15], [28], psychological variables were recorded after both exercise conditions. Ten minutes prior to each training session, the FS, FAS and POMS questionnaires were completed. Five minutes after the end of each activity, the participants responded to the FS, FAS, and POMS questionnaire again. Due to the time required to respond the FS, FAS, and POMS questionnaire, the PAES scores were elicited only 10 minutes after the activity ended.
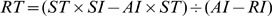
(1)Where

RT - recovery time (s); ST - stimulus time (s); SI - stimulus intensity (%); RI - recovery intensity (%); and AI - average intensity (%)

### Statistical Analysis

After assessing the normality of the data using the Shapiro-Wilk test, parametric analyses were performed. To compare the descriptive data of the HIT and CT sessions (average VO_2_, average HR, duration, and average VCO_2_) and the PAES scores, we used a paired t-test. The training sessions were divided into quintiles (Q1 to Q5), and the average value of each psychological variable corresponding to each quintile was used for the analysis. To determine the effect of the training condition (HIT or CT), the effect of time (pre to post exercise session) and their interaction (condition×time) on the psychological responses (FS, FAS, RPE, POMS), we used a two-way analyses of variance (ANOVA) with repeated measures. For the post hoc analysis, the *P* value was corrected for FS and FAS (.007), for the CR10 scale (.01) and for the POMS questionnaire (.025). To determine the magnitude of the differences between HIT and CT, we used an effect size analysis with CT as the reference condition. The effect size was interpreted as suggested by Hopkins [29] and was defined as follows: <.20, trivial; .21–.60, small; .61–1.20, moderate; 1.21–2.0, large; 2.21–4.00, very large; and >4.00, nearly perfect. The data from the FS and FAS were also represented in the circumplex model [9], which described the affective state with respect to activation (high and low) and valence (positive and negative). Analyses of significance were performed using GraphPad Prism v. 5.0 (GraphPad Software, San Diego, USA) with a significance level of *p*≤.05, and the effect size analyses were performed using Stata software v. 12 (StataCorp LP, College Station, USA).

## Results

### Descriptive results of training sessions

In the HIT condition, the average number of stimuli performed was 6.6 (*SD* = 1.7), with a recovery time of 57 seconds (*SD* = 10) between stimuli. The average duration of a CT session was 23.9 minutes (*SD* = 3.2). In the HIT condition, eight participants who were not able to complete the exercise session due to fatigue dropped out before the end of the activity; thus, the average duration was 19.2 minutes (*SD* = 4.8), which was significantly lower than that of the CT condition (*t* = 3.71, *p* = .01, η^2^ = .49). The average percentage of VO_2_ was 71.9% (*SD* = 7.5%) in the CT condition and 73.3% (*SD* = 3.5%) in the HIT condition, and no significant difference was found (*t* = 1.04, *p* = .31, η^2^ = .07). The average percentage of the maximum HR was 80.4% (*SD* = 4.4%) in the CT condition and 88.1% (*SD* = 2.4%) in the HIT condition, resulting in a significant difference between the two conditions (t = 7.96, p<.001, η^2^ = .82). The average VCO_2_ was 2.71 L.min^−1^ (*SD* = .36) in the CT condition and 3.13 L.min^−1^ (*SD* = .53) in the HIT condition, and the difference between the two conditions was significant (t = 5.70, p<.001, η^2^ = .69).

### Psychological variables

#### RPE, FS and FAS

Significant main effects were observed for the RPE (interaction, *F* = 4.48, *p*<.01; time, *F* = 63.84, *p*<.001; and condition, *F* = 29.46, *p*<.001), FAS (interaction, *F* = 2.29, *p*<.01; time, *F* = 38.15, *p*<.001; and condition, *F* = 18.65, *p*<.001), and FS (interaction, *F* = 4.93, *p*<.001; time, *F* = 22.89, *p*<.001; and condition, *F* = 7.92, *p*<.01). We observed significantly higher values for the RPE and FAS during HIT, whereas the FS values were lower during HIT. The significant interaction observed indicated that the RPE and FAS scores increased more over time, whereas the FS scores decreased more over time during HIT. The comparison between HIT and CT for each quintile is reported in Table 2.

**Table 2:** Comparison of psychological variables between HIT and CT for each quintile of exercise sessions.

Variables	Exercise session				*p*	*t*	Effect size (CI_95%_)
	HIT		CT				
	M	SD	M	SD			
FS							
Pre	2.07	2.55	2.00	2.24	>.05	.08	.03 (−.69, .74)
Q1	1.47	1.88	2.33	1.72	>.05	1.07	−.48 (−1.20, .25)
Q2	0.70	2.52	1.90	1.42	>.05	1.48	−.59 (−1.32, .15)
Q3	−0.27	2.86	1.40	2.10	>.05	2.06	−.67 (−1.40, .07)
Q4	−2.17	2.49	1.27	2.47	<.001	4.24	−1.39 (−2.19, −.58)
Q5	−2.67	2.64	0.80	2.54	<.001	4.28	−1.34 (−2.14, −.54)
Post	2.60	1.68	3.6	1.06	>.05	1.23	−.71 (−1.45, .03)
FAS							
Pre	2.47	1.55	2.47	1.36	>.05	.00	.00 (−.72, .72)
Q1	4.33	1.18	3.00	0.93	<.01	3.39	1.25 (.46, 2.04)
Q2	5.03	1.03	3.47	1.17	<.001	3.99	1.42 (.61, 2.22)
Q3	5.27	0.96	3.97	0.97	<.01	3.31	1.35 (.55, 2.15)
Q4	5.53	0.72	4.20	1.03	<.01	3.39	1.50 (.68, 2.31)
Q5	5.67	0.62	4.20	0.94	<.01	3.73	1.85 (.68, 2.31)
Post	3.53	1.25	2.47	0.99	<.05	2.71	.94 (.18, 1.70)
RPE							
Q1	4.37	2.23	1.77	0.86	<.01	3.38	1.54 (.72, 2.36)
Q2	5.80	2.15	2.9	1.10	<.01	3.77	1.70 (.86, 2.54)
Q3	7.47	2.55	3.92	1.99	<.001	4.61	1.55 (.73, 2.38)
Q4	8.83	2.31	4.67	2.51	<.001	5.41	1.72 (.88, 2.57)
Q5	9.67	1.95	4.83	2.62	<.001	6.28	2.10 (1.19, 3.00)

CI_95%_ - confidence interval; effect size classifications must be interpreted as <.20, trivial; .21–.60, small; .61–1.20, moderate; 1.21–2.0, large; 2.21–4.00, very large; and >4.00, nearly perfect.

#### POMS and PAES

We observed a significant time effect only for tension (*F* = 5.82, *p*<.05) and significant interaction and time effects for fatigue (interaction, *F* = 9.77, *p*<.01; and time, *F* = 15.74, *p*<.001), with a reduction in tension and an increase in fatigue after both exercise conditions. Although we observed an increase in fatigue in both conditions, the significant interaction indicated that the HIT condition resulted in greater increases in fatigue. The comparisons between HIT and CT for each POMS factor are reported in Table 3. There were no differences (*t* = .28, *p* = .779, η^2^ = .005) in PAES scores between the conditions (HIT, *M* = 97.8, *SD* = 17.3; and CT, *M* = 96.2, *SD* = 16.7).

**Table 3:** Comparison of POMS between HIT and CT.

POMS factors	Exercise session				*p*	*t*	Effect size (CI_95%_)
	HIT		CT				
	M	SD	M	SD			
Pre							
Tension	5.4	4.2	5.1	2.7	>.05	.23	.08 (−.64, .79)
Hostility	1.6	2.1	1.3	1.9	>.05	.00	.13 (−.58, .85)
Fatigue	3.7	4.4	3.6	2.3	>.05	.04	.02 (−.70, .74)
Vigor	13.0	4.8	11.3	4.8	>.05	.94	.35 (−.37, 1.07)
Confusion	3.7	2.1	5.0	1.7	>.05	1.53	−.67 (−1.41, .07)
Depression	1.1	1.8	1.3	1.9	>.05	.32	−.11 (−.82, .61)
TMD	102.5	10.2	105.0	8.1	>.05	.64	.27 (−.99, .44)
Post							
Tension	3.9	2.3	3.5	2.8	>.05	.35	.15 (.37, 1.92)
Hostility	1.0	1.5	.6	1.2	>.05	.63	.44 (−.29, 1.16)
Fatigue	8.7	4.9	4.2	2.5	<.01	3.30	−.39 (−1.11, .34)
Vigor	13.2	4.4	11.1	5.2	>.05	1.20	.00 (−.72, .72)
Confusion	3.7	2.9	4.7	2.0	>.05	1.21	.18 (−.53, .90)
Depression	.7	1.2	.7	1.5	>.05	.00	.09 (−.62, .81)
TMD	104.9	14.0	102.7	9.4	>.05	.56	.37 (.07, .67)

CI_95%_ - confidence interval; effect size classifications must be interpreted as <.20, trivial; .21–.60, small; .61–1.20, moderate; 1.21–2.0, large; 2.21–4.00, very large; and >4.00, nearly perfect.

#### Circumplex model

**Figure 1:**
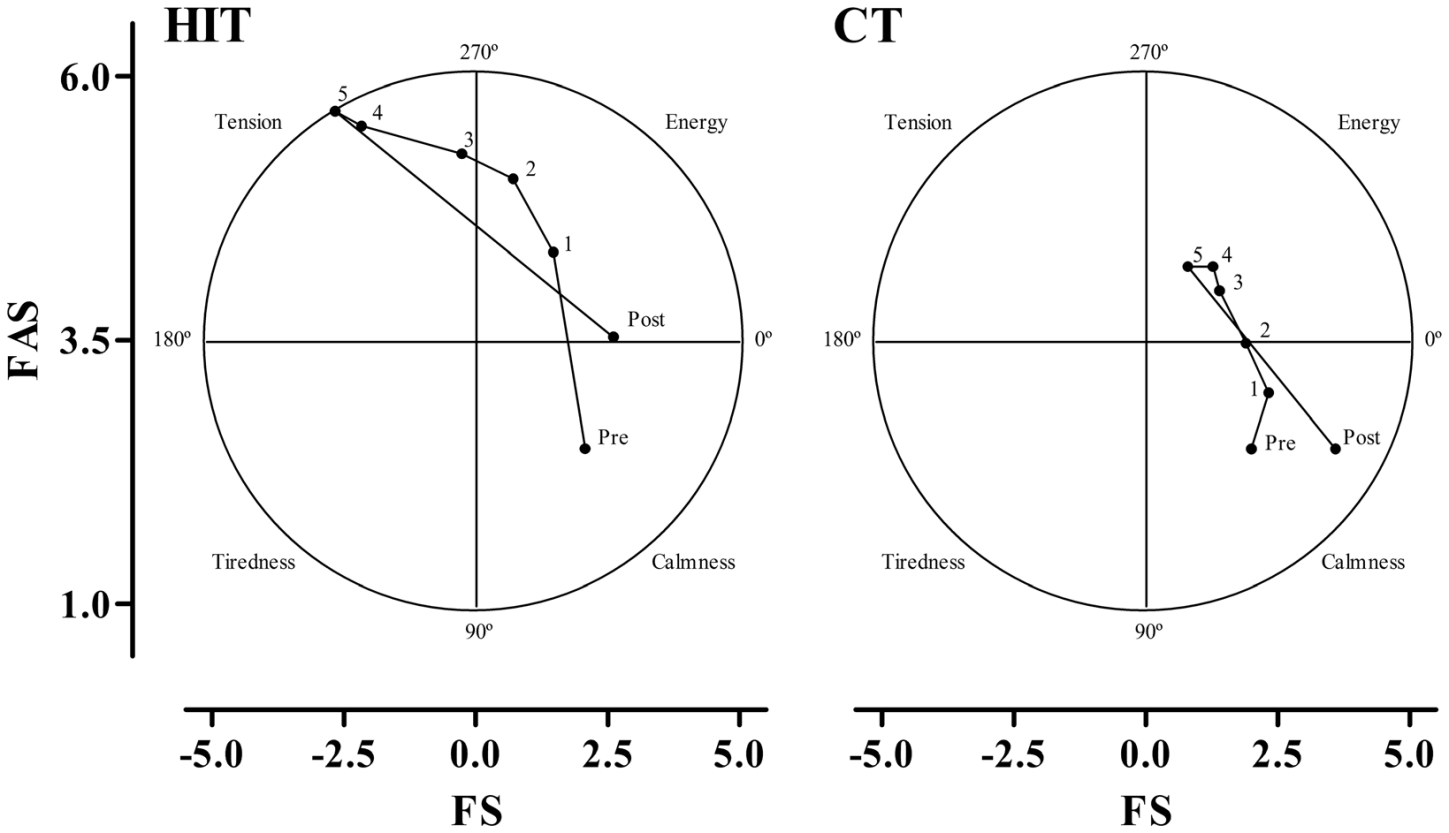
Circumplex model to CT and HIT sessions. HIT - high intensity interval training; CT - continuous training; FS - Feeling Scale; and FAS - felt arousal scale.

We observed differences in the patterns of the circumplex model. In the CT condition, the participants ranged from a sense of calmness (quadrant 1 - 0° to 90°) before the exercise session and in quintiles 1 and 2 to a sense of energy (quadrant 4 - 270° to 0°) after the exercise session and in quintiles 3, 4 and 5 (Figure 1). In the HIT condition before the exercise session, the participants ranged from a sense of calmness (quadrant 1 - 0° to 90°) to a sense of energy (quadrant 4 - 270° to 0°) in quintile 1 and a sense of tension (quadrant 3 - 180° to 270°) in quintiles 3, 4 and 5. After the HIT session, a sense of calmness was observed (quadrant 1 - 0° to 90°). All of the results are presented in Figure 1.

## Discussion

The objective of the present study was to compare the psychological responses to HIT and CT. To the best of our knowledge, previous studies investigating these psychological responses only addressed the pre- and post-exercise session time points. Moreover, basic affect was not previously investigated in these studies [7], [8]. We observed negative feeling scale responses in HIT compared to CT during and after the exercise session, as assessed by the circumplex model, but no differences were found in the post-activity PAES scores.

Our findings differed from the results observed by Bartlett et al. [7], who found that participants reported more enjoyment after HIT. It is important to consider that the FS and FAS are related to basic affect, whereas the PAES is related to an emotional state; in addition, the time of data collection was different for each variable (FS and FAS at 5 minutes post-exercise session and PAES at 10 min post-exercise session). Future studies should investigate the influence of different emotions on the adoption of physical activity. Moreover, differences in the methodological procedures may explain the divergent results. Bartlett et al. [7] used a prescription strategy of 3-minute intervals at a stimulus intensity of 90% of VO_2Peak_ and a 3-minute recovery period at an intensity of 50% of VO_2Peak_; both training conditions had an average intensity of 70% of VO_2Peak_. The training sessions utilized in our study were set at an average intensity that was 15% below the metabolic threshold. Despite the equality in VO_2_ values attained during the activities, the recovery period appears to have been insufficient to provide positive affective responses during the HIT sessions. Under these conditions, over 50% of the participants were unable to finish the HIT session. Although it was not the focus of this study, this is an important fact because self-efficacy may be negatively influenced in cases of participant dropout. It is possible that HIT sessions with longer recovery periods would provide better affective responses than the HIT sessions used in the present study. Future studies should consider the sprint ability of participants to confirm that they will be able to perform various tasks at high intensity.

Thus, disregarding the training method and considering that the average intensities in both studies (the present study and Bartlett's study) were very similar, at approximately 70% of VO_2Peak_, the affective response seems to have been influenced by the magnitude of the stimulus intensity and, consequently, by the predominant metabolic pathway engaged by the exercise. Following the Dual-Mode Theory, interoceptive factors seem to negatively influence affective responses to activities with intensities above the metabolic threshold [11]. This theory is widely recognized for CT but has not previously been demonstrated for HIT. Our study also confirmed this theory for HIT, demonstrating that an extreme increase in physiological responses during a stimulus resulted in an extreme decrease in pleasure during the activity independent of the recovery periods. It is possible that other HIT configurations with greater recovery periods could result in positive affective responses, and this hypothesis should be tested in future studies. The Dual-Mode Theory may also explain the positive rebound effect observed after exercise, with relatively larger increases in pleasure after HIT, which are possibly modulated by the magnitude of the negative perceptions during exercise [30]. The opponent process theory [31], postulates that after every affective perception (pleasant or unpleasant), an opponent process occurs. Thus, according to this theory, a feeling of pleasure can occur after an aversive stimulus or stress, which can activate the reward system and can then lead to a repetition of that stimulus. The increased production of neuromodulatory substances such as anandamide, dopamine, serotonin and endorphins may be associated with decreased anxiety and increased pleasure after intervals of intense stimulation [32]. The learning theory postulates that immediate affective responses should be better predictors of future exercise than the affective responses observed after the exercise session [33]. However, this hypothesis was not objectively investigated, and it is not well known whether individuals choose to continue to engage in physical activities based on perceptions experienced during or after exercise. Future studies should investigate this issue.

Some studies [28], [34] have used the strategy of equalizing the total work for different continuous interventions. Blanchard et al. [34] found no changes in affective responses based on the training condition. However, Kilpatrick et al. [28] demonstrated that higher intensities could generate negative feeling scale responses, even if the amount of work performed was equal. Regardless of the equalization of training with respect to total work or average intensity, activities that rely heavily on anaerobic metabolism result in negative pleasure and high arousal. Negative pleasure could induce exercise dropout and may be considered a negative result; on the other hand, high arousal may indicate better vigilance-sustained attention; therefore, it is not necessarily a negative result. It is possible that other strategies for equalizing the exercise intensity (e.g.: normalized power) provide better feeling scale responses.

Higher values for the average HR, irrespective of the average VO_2_, are associated with a greater sense of effort and negative feeling scale responses. Other models of HIT should be tested to determine the relationship between such models and patterns of affective responses, similar to relationship that has already been postulated for CT [28], [30], [35]. A combination of both HIT and CT would most likely be better to attain the expected benefits of exercise. However, it is possible that allowing participants to select the training method (HIT or CT) will provide better psychological responses [36]. Other factors, such as personal goals and an agonistic profile, may influence the decision-making for the selection of the training method. For example, the agonistic profile may influence the preference of individuals for exercise sessions with more or less suffering [37].

The lack of lactate monitoring during the exercise sessions was a limitation of this study because it was not possible to determine the influence of the acid-base balance during these activities. However, the VCO_2_ responses enabled us to infer the metabolic changes experienced by our participants. For the HIT condition, the affective responses were not recorded after the recovery period; they were only recorded after the stimulus. Although we believe that the response of the variable would be similar to what we observed, it is possible that this timing generated more negative responses during HIT. However, consecutive application of the scales over short interval periods could have induced reactivity in the participants, which may have altered their performance due to the awareness of being observed [38]. Although these tests occurred in a laboratory (which reduces the external validity), this study recorded the affective responses during a HIT session. Considering that HIT training and the measurement of affective responses have been consistently recommended in the literature to be used in a practical environment to promote health benefits and exercise adherence, the measurement of affect during HIT increases the external validity of the study.

In summary, CT performed at an intensity below the RCP results in positive affective responses compared with HIT at 100% of VO_2Peak_ with a short recovery time, which has a higher dependence on anaerobic metabolism even when performed at the same average intensity as that of the CT session. Despite the equalization of the average intensity (15% below of RCP) for both conditions, the dependence on anaerobic metabolism seems to be the determinant of psychological responses in HIT. Our results suggest that HIT should be used with caution regarding affective responses. However, we encourage future investigations regarding the effects of different interval training configurations. It is possible that HIT sessions with lower dependence on anaerobic metabolism induce better affective responses, as already demonstrated in different continuous training sessions [15], [30]. Considering the wide range of configurations for HIT sessions, it would be innaccurate to assert that CT is superior to HIT in providing better affective responses. Additional studies should investigate affective responses during other HIT configurations.
